# Ligation of Carbon Monoxide at Iron and Cobalt Single-Metal-Atom Sites: Competition for the +1 Oxidation State in a Surface-Confined Organic Network

**DOI:** 10.1021/acs.jpcc.6c04980

**Published:** 2026-09-15

**Authors:** A. Namar, B. Roondhe, S. Baronio, M. De Col, M. Jugovac, M. Scardamaglia, P. Giannozzi, E. Vesselli

**Affiliations:** † Department of Physics, University of Trieste, via A. Valerio 2, Trieste 34127, Italy; ‡ Department of Mathematics, Computer Science, and Physics, University of Udine, Udine 33100, Italy; § Department of Materials Science and Engineering, KTH the Royal Institute of Technology, Stockholm SE-100 44, Sweden; ∥ MAX IV Laboratory, Lund University, Lund 22100, Sweden; ⊥ CNR-IOM − Istituto Officina Dei Materiali, SISSA, Trieste 34136, Italy; # CNR-IOM - Istituto Officina Dei Materiali, S.S. 14 Km 163.5, Area Science Park, Basovizza, Trieste 34149, Italy; ∇ Center for Energy, Environment and Transport Giacomo Ciamician, University of Trieste, Trieste 34127, Italy

## Abstract

Cobalt coordination to a FeTPyP monolayer on graphene yields a two-dimensional FeTPyP–Co metal-organic network, stabilizing highly reactive Fe­(I) sites within a biomimetic coordination architecture. The assembly process triggers an electronic reorganization that activates the pristine Fe­(II) centers, enabling competitive CO ligation. A thorough synergistic investigation combining *in situ* photon-in/photon-out and photon-in/electron-out methods with density-functional theory simulations is presented. Distinct vibronic fingerprints reveal the complexity of the system, with anharmonic hot-band features associated with CO ligation at Co sites and a red-shifted mode uniquely assigned to CO ligation at Fe sites. Spectroscopic and theoretical results consistently indicate pronounced charge transfer and oxidation-state changes induced by adsorption. The bonding nature is site-specific, with linear CO at Co and tilted coordination at Fe. Quantitative pressure-dependent measurements uncover weak, fully reversible adsorption at room temperature, highlighting dynamic ligand-exchange processes and mild anti-cooperativity driven by indirect long-range, network-mediated interactions.

## Graphical abstract

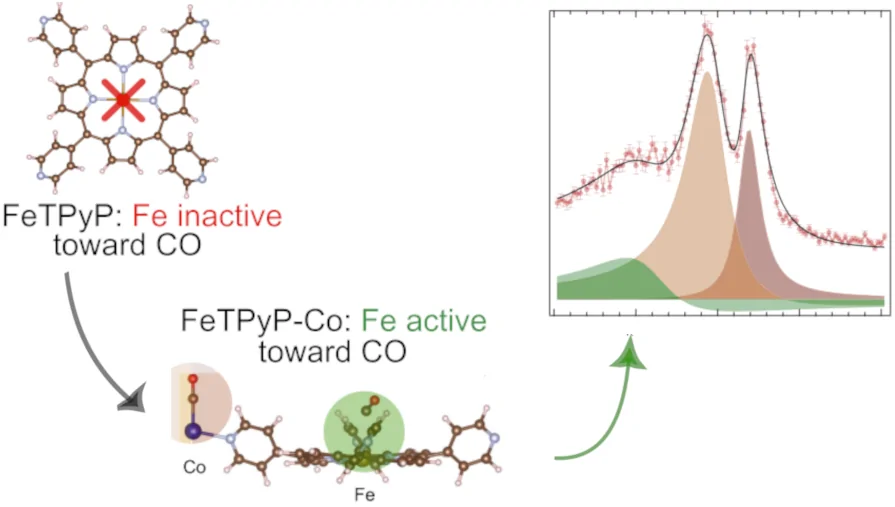

## 1 Introduction

Historically, adsorption of carbon monoxide has been extensively investigated in surface science since the earliest UHV studies,^1^ with the aim of providing fundamental insight into adsorption geometries and metal–adsorbate bond-formation mechanisms and, ultimately, developing more efficient catalysts for CO-related reactions. The chemical simplicity of this small molecule, combined with its stable adsorption configuration, typically the on-top (terminal) geometry, in which the carbon atom binds directly to the metal surface, makes CO a highly sensitive molecular probe for studying the properties of adsorption (surface science) and ligation (biochemistry) sites. Indeed, the internal C–O stretching frequency and the charge transfer affecting both core levels and molecular orbitals can be exploited to determine the geometry, coordination, and local electronic configuration of the adsorption site. In addition, the CO dipole moment makes it easily detectable by means of dipole-sensitive experimental methods, such as infrared reflection absorption spectroscopy (IRRAS) and infrared-visible sum-frequency generation (IR-Vis SFG) spectroscopy.^2–5^

Recently, increasing focus on the biochemistry of natural systems has fostered the investigation of artificial, biomimetic reaction centers specifically tailored for the activation and conversion of carbon monoxide.^6^ In particular, CO ligation at Fe centers is the first step for its conversion to hydrocarbons in natural nitrogenase systems,^7^ despite the fact that they are primarily dedicated to the conversion of N_2_ to NH_3_. This chemical flexibility is possible since the reactions take place at an FeS cluster, where a dynamic Fe coordination configuration environment is available,^8^ offering extended tuning of the local geometry, electronic density of states, and spin configuration. This allows charge delocalization, with local modulations of the nominal Fe oxidation state at different sites of the cluster around a +2 average, extending down to Fe­(I) oxidation state, yielding what is known as metal redox disproportionation.^7^ For this reason, the stabilization of the rare Fe­(I) state available for reaction has been addressed as a strategic development step in the design and synthesis of novel functional catalytic materials.^9,10^ To this purpose, we recently exploited a self-assembly strategy of porphyrin tectons on graphene (Gr) to synthesize and characterize a number of M_1_TPyP-M_2_ metal-organic networks with M_1,2_ = *, Fe, Co, Mn (* = empty site),^2,11–16^ starting from previous theoretical predictions and first experimental proofs.^17–22^ In particular, we succeeded in stabilizing Fe­(I) species in an FeTPyP-Co/Gr two-dimensional (2D) network.^16^

When cobalt adatoms are deposited on a FeTPyP/Gr/Ir(111) monolayer kept at room temperature, a substantial molecular rearrangement takes place. The interaction between Co and the pyridyl nitrogen atoms leads to tetra-coordination of Co by adjacent FeTPyP tectons and drives the formation of a well-ordered FeTPyP-Co coordination network (Figure S1). When a local Co:FeTPyP coverage ratio equal to 1.0 is reached, the initially close-packed FeTPyP superstructure evolves toward a structure that is best described by a (2 × 2), nearly square unit cell, with *a* = *b* = 3.9 ± 0.1 nm and α = 91.0° ± 1.9°. The periodicity is associated with an orientational modulation of the pyridyl moieties, which are alternately tilted clockwise and anticlockwise. While elemental Co is oxidized to Co­(I) upon coordination, the porphyrinic Fe center is reduced from Fe­(II) to Fe­(I). The latter state is found to be chemically metastable at room temperature (RT) even under ultra-high vacuum (UHV) conditions, being sufficiently stabilized by the network to be detected but highly reactive due to its peculiar electronic configuration. Similar MN_4_ centers (M = Fe, Co, Mn) were found to exhibit interesting novel properties associated with CO ligation. Long-lived excitons generated by impinging visible light were reported, originating from coherent singlet-fission mechanisms taking place at room temperature.^3^ We also observed vibrational excited states, namely hot bands, deviations from Langmuir isotherms due to coverage-dependent interactions,^23,24^ and specific, tunable reactivity properties of the single metal atoms.^12^ As discussed above, these 2D coordination networks act as biomimetic systems by reproducing the local coordination environment of single metal atoms in natural proteins, including both the square-planar nitrogen tetra-coordination and the axial “surface trans-effect” known as “fifth ligand”, provided in this case by the graphene support.^25^ In the following, we report a combined experimental and computational investigation of CO ligation to an FeTPyP-M (M = *, Co) network on graphene. As mentioned above, external coordination of Co to the peripheral pyridyl moieties shifts the oxidation state of the macrocyclic Fe atoms from +2 to +1, thereby activating these sites toward CO ligation. We carefully investigate the saturation curves as a function of the Co loading, quantitatively evidencing the role of (anti-)­cooperative mechanisms mediated by the organic network. These results are obtained using two *in situ* experimental methods, such as Ambient Pressure X-ray Photoelectron Spectroscopy (AP-XPS) and IR-Vis SFG, allowing for a detailed characterization of the electronic and vibronic structures, respectively, from UHV conditions up to the mbar pressure range.

## 2 Methods

### 2.1 Sample Preparation

The Ir(111) single crystal was cleaned by standard Ar^+^ sputtering (room temperature, *E_k_* = 2 keV) and annealing (1250 K) cycles, including intermediate treatments in an oxygen background (1 × 10^–7^ mbar, 570–1100 K) to remove residual surface and intercalated carbon contamination. A single-layer graphene (Gr) sheet was grown by thermal decomposition of ethylene.^26,27^ The Gr crystallinity was checked in reciprocal space by Low-Energy Electron Diffraction (LEED), which showed sharp Ir, Gr, and moiré diffraction patterns without rotational domains. As in our recent work,^16^ FeTPyPs (FeTPyPCl = 5,10,15,20-tetra­(4-pyridyl)­21H,23H-porphyrin Fe­(III) chloride, Frontier Scientific; purity > 95%) were then deposited *in vacuo* (low 10^–10^ mbar) upon sublimation from a hot boron nitride crucible with typical deposition rates in the order of 0.03 ML/min, after prolonged outgassing (500 K, nightlong) of the evaporator to remove both residual organic contaminants and the Cl ligand.^2,21,28,29^ FeTPyPs were deposited with the sample held at 500 K and a crucible temperature of about 600 K. Throughout the manuscript, we calibrate the surface coverage with respect to a complete single layer of FeTPyP molecules self-assembled on Gr/Ir(111).^11^ Thus, 1 ML of FeTPyPs in our notation corresponds to about 0.01–0.02 ML coverage with respect to the underlying Ir-termination unit cell (porphyrins per surface Ir atom). Deposition of single Co adatoms was achieved by sublimation from a resistively heated, pure Co wire (Goodfellow, 0.125 mm diameter, 99.99% purity), with the substrate kept at 400 K for best ordering and to avoid Co intercalation under the Gr sheet.^30^

### 2.2 IR-Vis SFG Spectroscopy and Data Analysis

Infrared-Visible Sum Frequency Generation (IR-Vis SFG)^31,32^ vibrational spectroscopy measurements were performed in the laboratories of the Department of Physics of the University of Trieste, hosted at CNR-IOM.^33^ A dedicated UHV system with a base pressure of 5 × 10^–11^ mbar equipped with standard surface science preparation and characterization techniques, is directly coupled with a high-pressure cell for *in situ* IR-Vis SFG spectroscopy. The reactor is equipped with a gas system to handle the reactants’ pressure in the 10^–9^–10^+3^ mbar range. The inlet and outlet of the laser beams are provided by UHV-compatible BaF_2_ windows. The excitation source (Ekspla, 1064 nm, 30 ps, 50 Hz) delivers a 532 nm (2.33 eV) visible beam and IR radiation, tunable in the 1000–4500 cm^–1^ range, obtained via second-harmonic and parametric generation. The raw spectra were normalized to the impinging infrared and visible excitation intensities to account for the energy-dependent IR intensity and time-dependent source intensity fluctuations. The normalized SFG spectra were then analyzed by least-squares fitting to a widely adopted parametric effective expression of the nonlinear second-order susceptibility,^31,34^

1 $$\frac{I_{SFG}\left(\omega_{IR}\right)}{I_{Vis}I_{IR}\left(\omega_{IR}\right)}\propto \left|A_{NRes}+\underset{k}{\sum }\frac{A_{k}e^{i{\Delta \Phi }_{k}}}{\omega_{IR}-\omega_{k}+i\Gamma_{k}}\right|2$$

where *A_NRes_*, *ω_IR_*, *A_k_*, Δ*φ_k_*, *ω_k_*, Γ_*k*_, are the non-resonant background amplitude, the impinging IR radiation frequency, and the k-th resonance amplitude, relative phase, frequency, and dephasing time-related width, respectively. The model well reproduces the observed line shapes, accounting for the resonant IR-Vis vibronic transitions and the nonresonant background, describing all the interference terms associated with the relative phases, and considering the Lorentzian broadening typical of a stimulated spectroscopic transition and related to the dephasing rate, which in turn stems from the excited-state lifetime and the elastic dephasing of the vibronic state.^35^ In the figures, we plot the normalized IR–Vis SFG signal intensity (markers with measured statistical error bars), together with the best-fit (lines) and the deconvolution of the resonances with their interference with the nonresonant background (color-filled profiles).^33^ We recall that multiplet solutions are possible due to the non-uniqueness of the parameter sets.^36^ Further details and examples can be found in our previous works.^12,16,33,37^ In the present study, all spectra were collected in a *ppp* polarization combination (SFG-visible-infrared).

### 2.3 Ambient-Pressure X-ray Photoelectron Spectroscopy (AP-XPS)

Photoelectron spectroscopy measurements, both in UHV and at ambient pressure, were carried out at the HIPPIE beamline (3 GeV ring) of the MAX IV synchrotron radiation facility in Lund (S). The soft X-ray beamline is designed to perform *in situ* and *operando* XPS experiments up to 30 mbar.^38^ The photon source is an APPLE-II-type elliptically polarizing undulator with full polarization control. The photon energy can be tuned over the 250–2200 eV range, yielding photon fluxes larger than 10^12^ photons/s. The beam size is about 60 × 25 μm^2^ (horizontal × vertical), determining a 100 × 25 μm^2^ spot at the sample due to a grazing incident angle. We selected photon energies of 1000, 750, and 514 eV for the Co and Fe 2*p*_3/2_, O 1*s*, and N 1*s* core levels, respectively. The Scienta Omicron HiPP-3 electron energy analyzer has a differential pumping stage and an electrostatic lens system that allow for AP operation at up to 30 mbar at a 0.3 mm nozzle diameter. The gas inlet system consists of eight independent gas lines, each equipped with its own mass flow controller, enabling fine-tuning of both pressure and gas flow. The gas composition of the reaction cell can be monitored using a quadrupole mass spectrometer. Further technical details about the setup can be found elsewhere.^38^

### 2.4 Density Functional Theory Calculations

All calculations were performed using spin-polarized density functional theory (DFT) as implemented in the Quantum ESPRESSO package.^39,40^ Exchange-correlation effects were described within the generalized gradient approximation using the Perdew–Burke–Ernzerhof (PBE) functional.^41^ Dispersion interactions, important for molecule–surface interactions, were included using the Grimme DFT-D3 correction scheme.^42^ On-site Coulomb interactions for the transition-metal (3*d*) states were treated using the DFT+U approach within the rotationally invariant formulation of Dudarev et al.^43^ A Hubbard U value of 3.0 eV was applied to Fe 3*d* states in both systems, while a value of 6.14 eV was applied to Co 3*d* states in the Co-containing system.

Core–valence interactions were treated using a combination of ultrasoft and projector augmented-wave (PAW) pseudopotentials from the PS Library.^44^ A kinetic energy cutoff of 60 Ry for the wavefunctions and 240 Ry for the charge density was employed based on convergence tests. Brillouin zone sampling was performed using a Monkhorst–Pack^45^ k-point mesh of (4 × 4 × 1) for the FeTPyP-Co/Graphene system (150 atoms). Methfessel–Paxton smearing with a width of 0.01 Ry was used to facilitate electronic convergence.

The simulation cell is orthorhombic for the bimetallic FeTPyP-Co/graphene system with lattice parameters *a* = 14.76 Å and *b* = 12.83 Å. Consistent with previous studies,^2,11,16,17,21,46,47^ this supercell configuration reliably captures the structural and spin-state characteristics of comparable porphyrin-based systems. A vacuum spacing of ∼20 Å was introduced along the surface normal to avoid spurious interactions between periodic images. Structural relaxations were carried out using the Broyden–Fletcher–Goldfarb–Shanno algorithm. All structures were considered converged when the forces on each atom were smaller than 10^–3^ Ry/Bohr. Spin-polarized calculations were performed with finite initial magnetizations on the transition-metal atoms to stabilize the magnetic states.

Bader charge analysis^48,49^ was used to quantify charge transfer between the adsorbed (CO) molecule and the active sites. Charge-density difference plots were generated to visualize electron redistribution upon adsorption according to

2 $$\Delta \rho =\rho_{total}-\rho_{substrate}-\rho_{CO}$$

where *ρ*_*total*_ is the density of the final complex, and *ρ*_*substrate*_ and *ρ*_CO_ are the densities of the isolated components at their optimized adsorbed geometries. The adsorption energy *E*_*ads*_ of CO was calculated as

3 $$E_{ads}=E_{CO+surface}-E_{surface}-E_{CO}$$

where *E*_CO+*surface*_, *E_surface_*, and *E*_CO_ correspond to the total energies of the adsorbed system, the clean substrate, and the isolated (CO) molecule, respectively.

## 3 Results and Discussion

### 3.1 Vibronic Characterization

The internal C–O stretching band of terminal (on-top) CO is generally observed in the 1800–2120 cm^–1^ range at metal surfaces, metallic nanoclusters, single metal atoms, and metal-organic molecules.^2,3,23,50–52^ The redshift, with respect to the gas-phase value at 2143 cm^–1^,^53^ originates from the weakening of the internal bond associated with charge transfer from the substrate to the π* molecular antibonding states, which is well described within the framework of the Blyholder model.^54,55^ Here, upon exposure of an FeTPyP/Gr/Ir(111) monolayer to carbon monoxide at room temperature, formation of the Fe­(II)-CO bond is not observed, at least up to the near-ambient pressure range (Figure S2). Instead, the IR-Vis SFG spectra collected in the 1850–2050 cm^–1^ stretching region in the case of the bimetallic FeTPyP-Co/Gr/Ir(111) metal-organic network show the growth of three well-resolved resonances (Figure 1). As already described in our recent work and summarized in the introduction,^16^ relevant geometric and electronic structure changes take place upon Co coordination to the terminal N atoms of the pyridyl moieties of the porphyrins, inducing a strong modulation of the reactivity of both the central Fe and the coordinated Co atoms. Under steady-state equilibrium conditions at room temperature in a 0.1 mbar CO, dynamic equilibrium between CO adsorption and desorption rates is achieved, which is dictated by both temperature and the CO background pressure.

**Figure 1:**
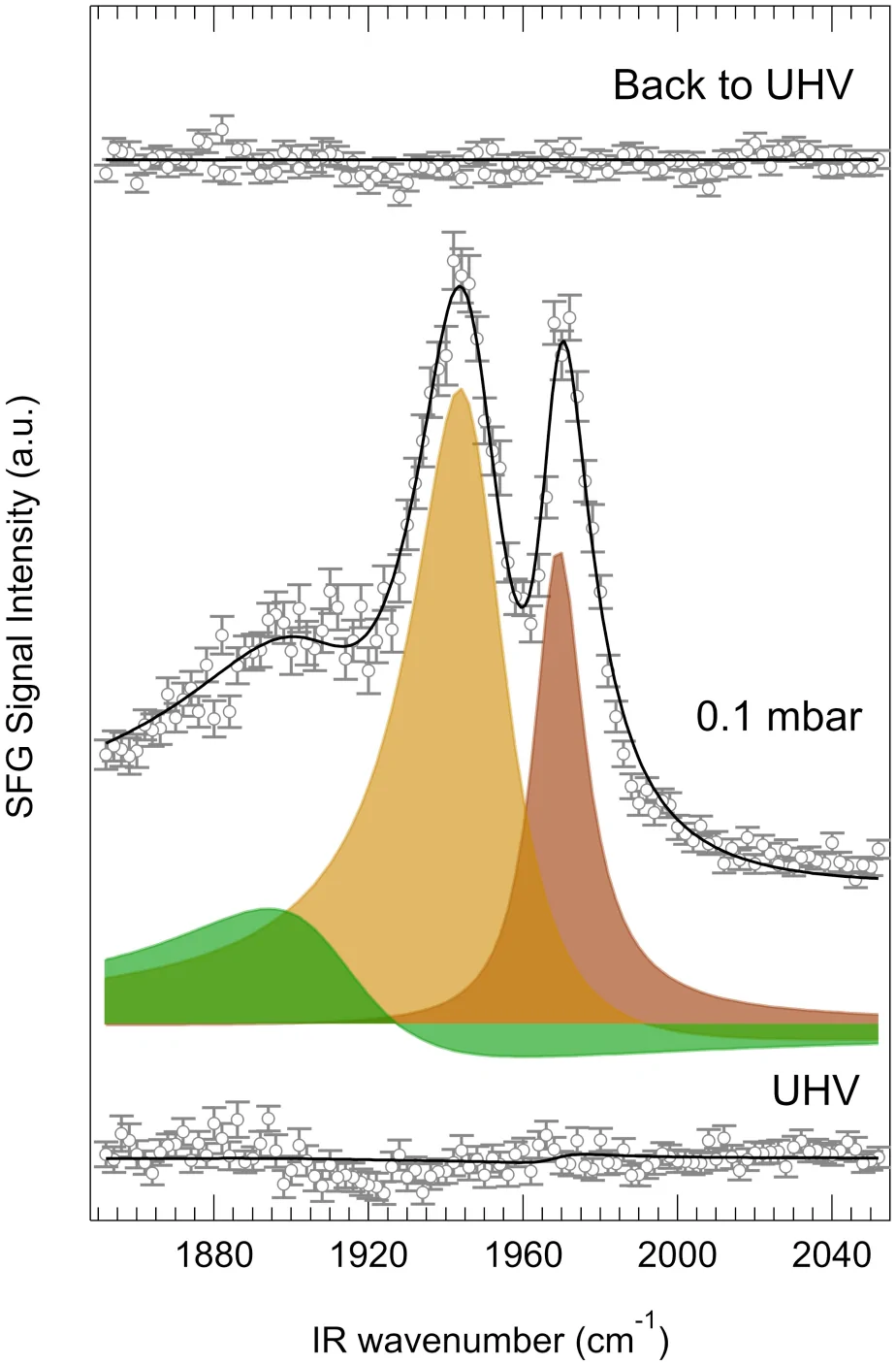
IR-Vis SFG spectra collected *in situ* in the C–O internal stretch region for the as-prepared FeTPyP-Co/Gr/Ir(111) system (bottom), in a 0.1 mbar CO background (middle), and after recovering UHV (top); normalized experimental data (markers) are reported together with the best fit (black lines) and the corresponding resonances (filled profiles); all spectra were collected in the *ppp* polarization combination and at room temperature.

Three intense vibronic resonances are observed at 1909, 1946, and 1969 cm^–1^, which reversibly disappear when UHV conditions are recovered. On the basis of previous results on similar bimetallic networks, namely CoTPyP-Co and MnTPyP-Co,^23,24^ we assign the latter two resonances to the 1→2 (hot-band) and 0→1 (fundamental) transitions of the vibrational ladder of the internal C–O stretching mode of CO adsorbed at the Co sites. Multiple resonances associated with a single vibronic mode can generally arise from non-equivalent adsorption sites or geometries,^56,57^ which usually induce shifts that are larger than 23 cm^–1^ observed here. CO adsorption at Co clusters originating from defects in the layer formation would instead contribute resonances above 2000 cm^–1^.^23,58^ Vibronic splitting due to excitonic coupling could also be considered, but it generally results in much smaller energy separations of a few cm^–1^.^3^ Finally, vibrational anharmonicity provides the most natural explanation. CO ligated to the Co single atoms can be regarded as a localized oscillator with an anharmonic interatomic potential. As a result, a resonance originating from the n → n + 1 transition occurs at progressively lower energy as n increases.^2,23,59^ Based on these considerations, the component at 1946 cm^–1^ is consistent with a hot-band transition, separated from the fundamental by 23 cm^–1^ (≈2.9 meV). Concerning the amplitude ratio between the two resonances, we find experimentally that it depends on the intensity of the incident IR beam, in agreement with our attribution. In SFG, the resonant amplitude scales with the population difference between the initial and final vibrational states. This population difference is influenced by the IR excitation intensity, which accounts for the observed behavior and further supports our assignment. We also recall that the n = 1→2 transition has a higher transition dipole moment, leading to a more intense resonant signal.^60^

Interestingly, a third resonance is clearly resolved at 1909 cm^–1^, which was not observed in our previous works on the similar CoTPyP-Co and MnTPyP-Co/Gr/Ir(111) systems,^23,24^ representing a peculiarity contributed by the Fe species. In a first approach, it could be indeed erroneously interpreted as the n = 2→3 transition of the CO@Co vibrational ladder. However, this assignment can be readily excluded on the basis of simple anharmonicity considerations. The spacing between this third feature (green envelope in Figure 1) and the hot band (yellow envelope) is 37 cm^–1^, which is larger than the energy spacing between the other two resonances. This is in contrast with the expected progressively decreasing separation between the rungs of the anharmonic vibrational ladder. Instead, a reasonable explanation considers that, upon Co coordination and formation of the metalorganic network (MON), a strong electronic reorganization takes place, affecting the Fe atoms, which are known to shift from the initial +2 to the rare +1 oxidation state,^16^ thereby enabling CO ligation. This hypothesis is confirmed by our AP-XPS measurements and DFT simulations, which demonstrate CO adsorption at both Fe and Co centers.

### 3.2 Electronic Structure Characterization

AP-XPS of Fe and Co 2*p*_3/2_ core levels, along with O and N 1*s* signals (Figure 2), were collected at room temperature before, during, and after CO exposure. The maximum CO pressure used for this experiment was 0.03 mbar: sufficiently high to ensure saturation of Fe and Co sites, as observed in the IR-vis SFG measurements, while minimizing the contribution of residual contaminants. In the bottom row of each panel in Figure 2, the core-level spectra of the as-prepared FeTPyP-Co layer collected in UHV are shown, in agreement with our previous findings.^16^ In the UHV spectrum, two small but evident components are ascribed to contaminant atomic O species bound to carbon (gray, 531.5 eV) and to the single-metal-atom sites (blue, 529.7 eV). These O species originate from the activation of oxygen-containing molecules (mainly water) in the residual UHV background by the highly reactive Fe­(I) states of the network (see ref 16 for further details).

**Figure 2:**
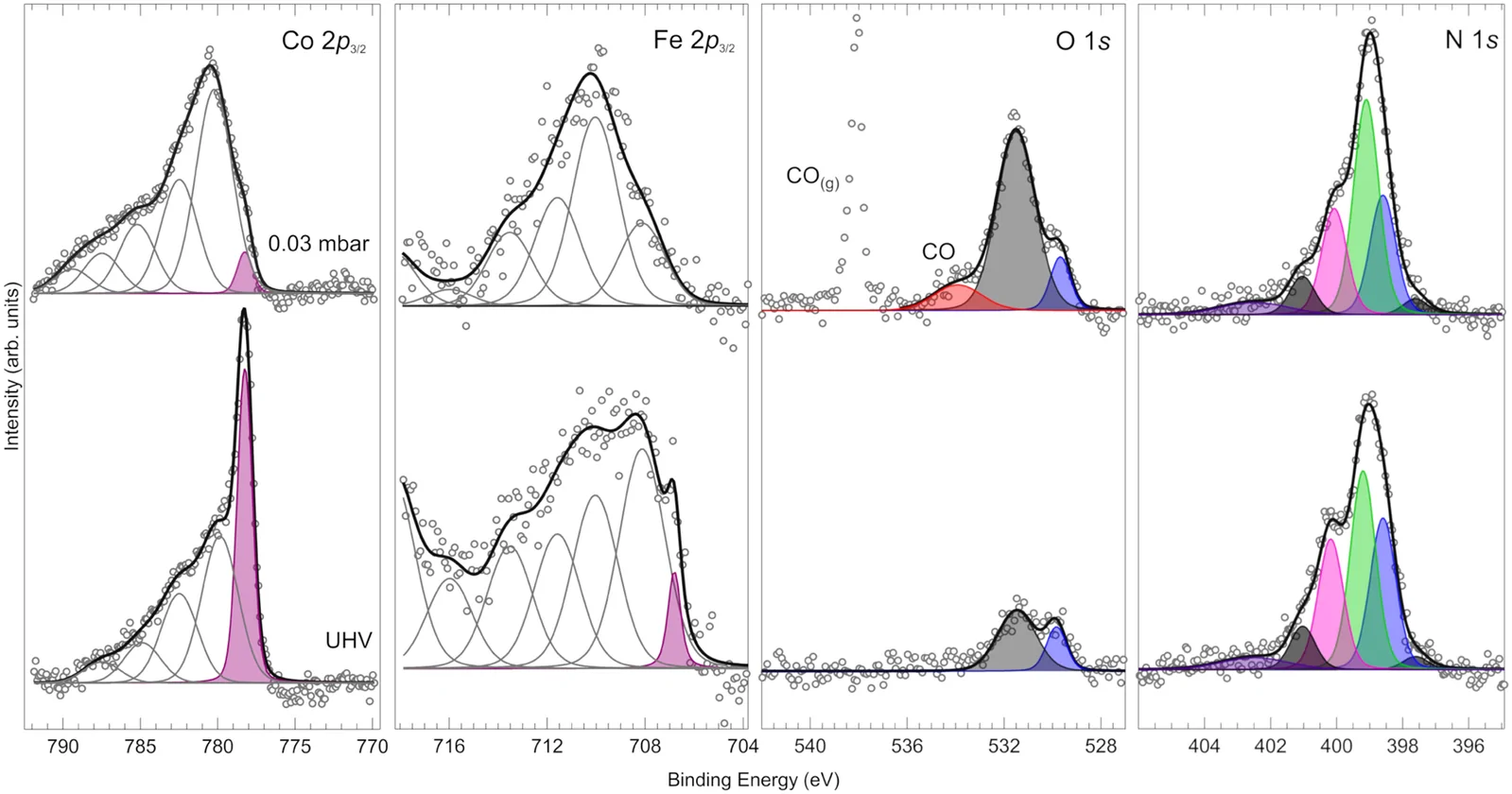
Synchrotron radiation AP-XPS spectra collected *in situ* for the as-prepared FeTPyP-Co/Gr/Ir(111) system (bottom) and in 0.03 mbar CO (top) at room temperature; from left to right, Co 2*p*_3/2_ (hν = 1000 eV), Fe 2*p*_3/2_ (hν = 1000 eV), O 1*s* (hν = 750 eV), and N 1*s* (hν = 514 eV).

Upon introduction of the CO background at room temperature, a stationary adsorption-desorption equilibrium is established, leading to readily observable modifications in the O 1*s* spectrum. While the CO gas phase contributes at 538.1 eV,^61^ a new component arises at 533.9 eV (red), which is attributed to terminal CO weakly bound to the metal sites,^62^ in line with previous AP-XPS studies of CO adsorption on a MnTPyP-Co/Gr layer.^24^ In agreement with the IR-Vis SFG data, this component vanishes upon recovering UHV conditions, further supporting our attribution to ligated CO (Figure S3). In parallel, the relative intensity of the carbon-bonded O species signal (gray) increases to nearly three times its initial value, most likely due to unavoidable background contamination. This increase is not observed for the O species bonded to the metal sites (blue), indicating that CO exposure contributes to reducing this component. Only minor, negligible modifications are observed in the N 1*s* region, where the profile of the pristine layer in UHV maintains its original shape during CO exposure, apart from small intensity modulations. We resolve here five distinct components at 399.2 and 400.2 eV (iminic and pyridinic N species, green and pink, respectively), 397.6 (black, iminic N), and 401.0 eV (pyridinic N), associated with porphyrins coordinated with Co clusters, and a high-binding-energy shake-up feature.^16^ However, the involvement of both Co and Fe metal species in CO ligation is revealed by the corresponding 2*p*_3/2_ core-level spectra. In the pristine layer, the Co spectrum reveals multiplet splitting components at 778.3, 779.9, 782.5, 784.9, and 787.5 eV, corresponding to a Co­(I) species, according to the Gunnarsson–Schönhammer (GS) screening model, which accounts for the observed low-energy, sharp feature (pink).^16,63,64^ Upon CO adsorption, the GS component in the Co 2*p*_3/2_ spectrum is reduced by ∼85%, whereas the Fe 2*p*_3/2_ spectrum shows complete quenching of the GS component, reflecting suppression of the charge-transfer mechanism responsible for this feature under UHV conditions. During CO exposure, the dominant feature is located at 780.2 eV, characteristic of Co­(II), while a new component emerges at 789.4 eV, possibly associated with multiplet splitting components of a higher oxidation state toward Co­(III).^65^ Also in the case of Fe 2*p*_3/2_, the intensity of the higher oxidation contributions (empty envelopes) is modulated, while the predominant feature shifts to 710 eV, consistent with a switch of Fe from the +1 to the +3 oxidation state.

The metal oxidation states inferred from the experimental data are further supported by spin-polarized DFT calculations using the PBE exchange-correlation functional, with Bader charge analysis used to quantify the electronic population at each metal center (Figure 3). Upon CO ligation, the calculated Bader charge for Co is ≈ 8.1 e^–^, consistent with Co­(III), while for Fe it is ≈6.8 e^–^, indicative of low-spin Fe­(III). The charge redistribution was calculated as the difference between the total charge density of the combined system (CO@FeTPyP-Co/Gr) and the sum of the charge densities of its isolated components, integrating over the in-plane coordinates of the unit cell for each z component in order to get the Δ­(*Z*) profile. In the figure, we superimpose the optimized geometry of the layer and ligated CO molecules (a) with the charge accumulation isosurfaces (b), where regions of electron accumulation are shown in yellow and regions of electron depletion in cyan, respectively, using an isovalue of ±0.076 e^–^/Å^3^. At Co sites, the CO ligand adopts an upright orientation, whereas at Fe sites it is tilted by 63° with respect to the normal to the surface. The calculated charge transfer amounts to 0.29 e^–^ from Co to CO and 0.049 e^–^ from Fe to CO. The C–O bond lengths are estimated as 1.15 Å on Co and 1.14 Å on Fe, compared to a gas-phase value of 1.13 Å, indicating that the CO molecule remains intact and does not dissociate upon adsorption.

**Figure 3:**
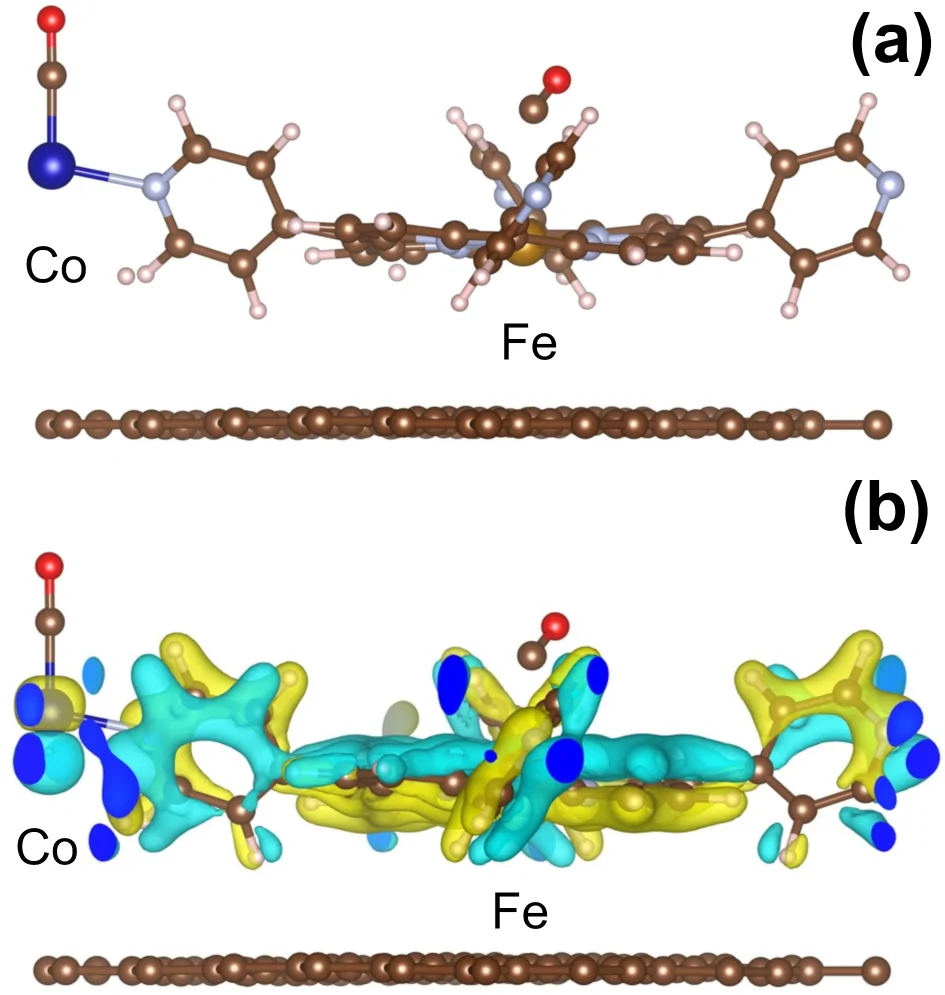
Results from the DFT simulations: (a) relaxed, optimized structure of the FeTPyP-Co network on graphene with CO ligated at both Fe and Co single-metal-atom sites (color code: C – brown, N – grey, H – white, O – red, Co – blue, Fe – gold); (b) charge density difference highlighting the electron redistribution taking place in the network upon CO ligation (color map: yellow and cyan represent isosurfaces of ±0.076 e^–^/Å^3^, respectively), while 0.29 (0.049) e^–^ are transferred to CO ligated at the Co (Fe) sites. The blue areas correspond to graphical artifacts arising from the finite size and periodic boundary conditions of the simulation cell.

### 3.3 Saturation Isotherms and Cooperativity

The extreme surface sensitivity of IR-Vis SFG enables a detailed *in situ* investigation of the adsorption process as a function of progressively increasing pressure, from UHV to saturation. It should be noted that, in the present system, saturation corresponds to only about 1% of a monolayer (ML), i.e., to the absolute coverage of the available single Fe and Co sites. This allows a quantitative investigation of the kinetics, evaluation of the possible role of cooperative mechanisms, and measurement of the coverage-dependent CO adsorption energy at Fe and Co. To probe the influence of the metal loading and, thus, of the geometry of the MON, three systems were prepared, each with different Co relative loadings corresponding to Co:Fe ratios of 0.04, 0.40, and 0.70. Co was kept well below the 1:1 ratio to exclude contributions from Co clusters, either coordinated to the pyridinic sites or to the residual bare graphene domains.^16^ In principle, the ligand coverage of a FeTPyP-Co/Gr/Ir(111) monolayer increases with the CO background pressure, being determined by the stationary equilibrium between adsorption and desorption processes. The CO stretching region in the 1850–2050 cm^–1^ range was monitored *in situ* as a function of the increasing CO pressure, from UHV to 0.1 mbar, spanning 8 orders of magnitude in pressure without loss of continuity. The uptake data are plotted in panels (a–c) of Figure 4, where the measured SFG intensity (color scale) is displayed as a function of both IR wavenumber and CO background pressure. In each of the three experiments, the SFG spectrum of the as-prepared layer is completely flat in the 1850–2050 cm^–1^ region under UHV conditions.

**Figure 4:**
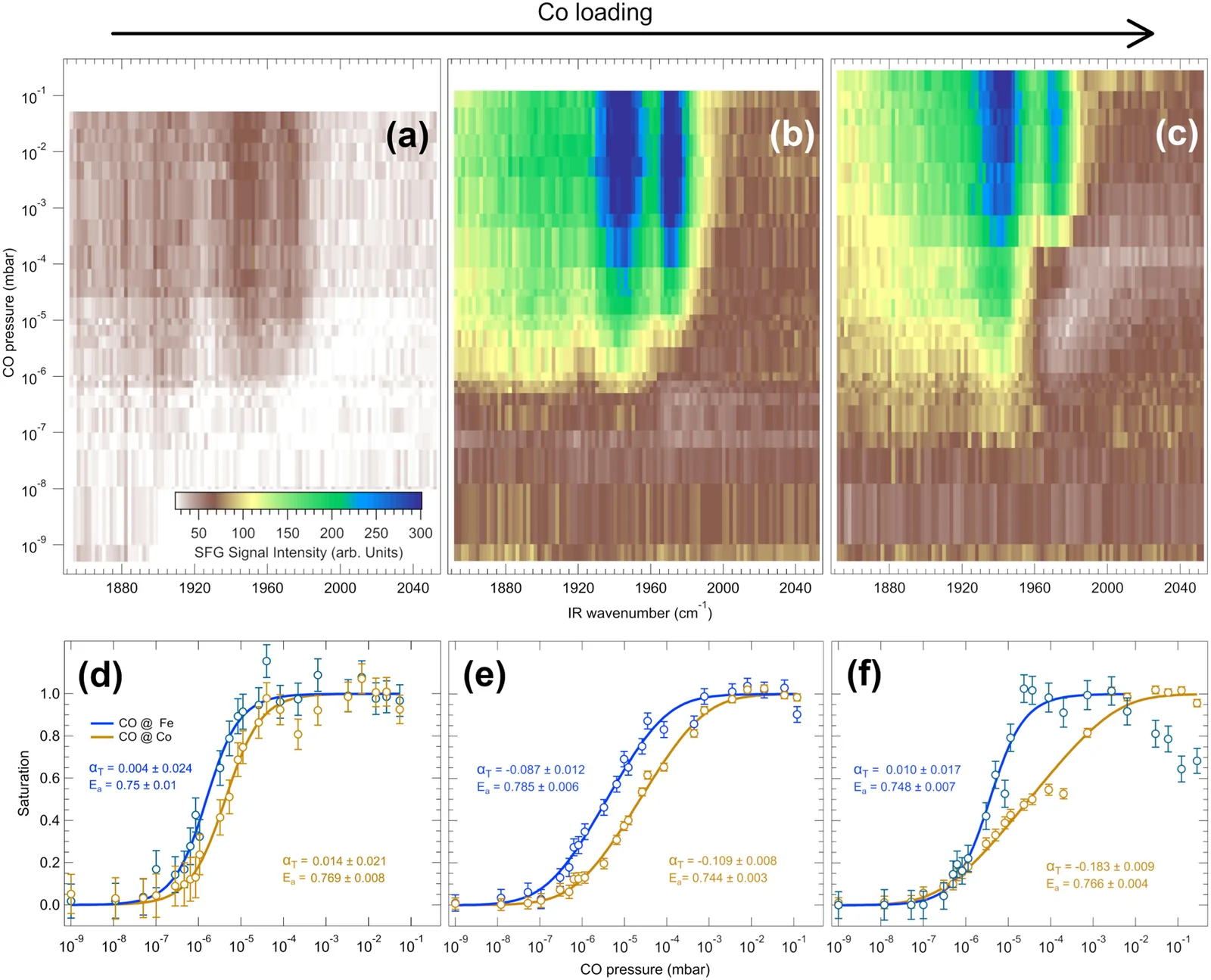
IR-Vis SFG spectra (a–c) were collected *in situ* at room temperature as a function of the (growing) background CO pressure, starting from the as-prepared FeTPyP-Co systems in UHV; the corresponding CO saturation curves for both sites (Fe – blue, Co – orange) as obtained from the resonances amplitudes are reported in panels (d–f), where the continuous lines indicate the best fit of the isotherms according to the Temkin model;^66,67^ the experiment was repeated for increasing Co loadings (see text for the definition): 0.04 (a,d), 0.40 (b,e), and 0.70 (c,f); all spectra were collected in the *ppp* polarization combination.

Upon introduction of a CO background at around 10^–8^ mbar, a weak signal starts to be detectable at 1909 and 1942 cm^–1^, progressively increasing with pressure and is associated with CO@Fe and the hot-band contribution from CO@Co atoms, respectively. A third resonance starts growing at 1968 cm^–1^ around 10^–6^ mbar, corresponding to the fundamental transition of CO@Co, with a phase that evolves along with the uptake. In all cases, the resonance associated with the internal C–O stretch of CO@Fe appears at a lower wavenumber than those associated with CO@Co, suggesting more coordinated adsorption, and is thus consistent with the DFT simulations, which indicate a bent terminal ligation configuration. This geometry affects the dipole coupling with the molecular layer, shifting its vibrational contribution toward lower wavenumbers relative to the terminal, vertical configuration of CO@Co.

Regarding the resonant amplitudes, it is worth recalling that the hot band of CO on Ru(0001) and Ir(111) was previously observed by means of broadband SFG,^59,60^ unlike in our scanning-mode case. In that configuration, the impinging IR beam has a bandwidth of several 100 cm^–1^, enabling hot-band excitation through absorption of two IR photons with different energies, resonant with both 0→1 and 1→2 transitions, respectively. SFG spectra obtained under such conditions provide a more accurate representation of the population ratio between the ground and first excited vibrational levels. By contrast, in a scanning-IR SFG configuration, the excitation is provided by a narrow IR pulse (of the order of 1 cm^–1^ width). Ideally, to study the hot band in a scanning configuration, the fundamental transition should be excited resonantly with a dedicated fixed-wavelength pump IR pulse, while performing the SFG measurement using a second IR beam. In our case, however, at each point of the spectrum the system is both pumped and probed by the same scanning-wavelength IR pulse, and the 0→1 and 1→2 transitions are excited by IR photons with the same energy. The hot band is populated and detectable only because of the intrinsic broad line width of the partially overlapping resonances. Consequently, an independent quantitative analysis of the single hot and fundamental states populations would not be physically meaningful, as their contributions cannot be fully disentangled. For this reason, in the case of CO ligation at Co sites and as the best proxy, we tracked the evolution of the sum of the resonant signals, keeping in mind that the signal amplitude is proportional to the state population (coverage). To simplify the spectral modeling and reduce the number of degrees of freedom, the data imaged in Figure 4a–c were fitted with globally optimized widths and phases. The phase of the CO@Co fundamental transition was allowed to vary to follow its pressure-dependent evolution. The saturation curves obtained from the best fit of the data plotted in Figure 4a–c are reported in panels d–f of the same figure. These profiles were obtained by plotting the resonance amplitudes as a function of the CO background pressure, along with error bars derived directly from the fit by propagating the experimentally measured uncertainties. The evolution of the resonance amplitude associated with CO ligation at the Fe (Co) sites is shown in blue (yellow). Fe sites are saturated earlier than Co sites, and the saturation pressure values depend on the Co loading. Interestingly, for the highest Co loading (Figure 4f), an anomalous trend is observed for the Fe sites: after reaching saturation at about 10^–4^ mbar, the site occupancy decreases above 10^–2^ mbar, while the Co sites approach saturation. This behavior may originate from a change in the electronic configuration of the layer, induced by CO saturation of the Co sites, which makes adsorption at the Fe sites less favored.

The uptake profiles were fitted with two independent models: the Temkin isotherm, an extended version of the Langmuir isotherm,^66,67^ and the Hill equation.^68–70^ Both models almost perfectly overlap and well reproduce the experimental data. Thus, for clarity, only the Temkin fit is displayed in the graphs (continuous lines), although both the Temkin and Hill models were optimized on the data. These models are widely used in catalysis, biochemistry, and pharmacology. The Temkin isotherm accounts for coverage-dependent adsorption energies, while the Hill equation provides insight into the degree of cooperativity, as a many-body effect arising from interactions between adsorption sites. We could evaluate the adsorption energy of CO at both Fe and Co sites by using the Temkin model. We note that the latter has been widely misused and cited in the literature in a dimensionally inconsistent form. Recently, significant effort has been made to clarify this issue and to recover its correct form and approximations.^66^ Here, we adopt the following expression, already applied to describe adsorption on metal porphyrins:^71^

4 $$E_{\mathrm{ads}}\left(\theta \right)=E_{\mathrm{ads}}^{0}\left(1+\alpha_{T}\frac{\theta }{\theta_{\mathrm{sat}}}\right)$$

where *E*_*ads*_(*θ*) is the (linearly) coverage-dependent adsorption energy, *E*_*ads*_^0^ is the initial (zero-coverage limit) adsorption energy, *α_T_* is the Temkin parameter, *θ* and *θ_sat_* are the coverage and saturation coverage, respectively. The approximation of a linear adsorption heat dependence on *θ* reflects the degree of (anti)-cooperativity of the process, with *α_T_* assuming values <0 (anti-cooperativity), = 0 (no cooperativity), or >0 (cooperativity). From the SFG amplitudes, we extract the evolution of the normalized coverage term *θ*/*θ_sat_*, obtained by normalizing the resonant amplitude of the C–O stretching mode to its saturation value.

In parallel, the Hill equation provides complementary insight into the degree of cooperativity between adsorption sites.^70^ Originally introduced by Hill more than a century ago to describe the cooperative binding of oxygen to hemoglobin,^68^ the model relates the fraction of occupied receptors to the ligand concentration as

5 $$\frac{\theta }{\theta_{\mathrm{sat}}}=\frac{p^{n}}{p_{0.5}^{n}+p^{n}}$$

where *θ*/*θ_sat_* represents the ratio between occupied and total available binding sites, *p* is the gas pressure, *p*_0.5_ is the pressure at which half of the receptors are occupied (half-saturation pressure), and n is the Hill coefficient.^70^ The latter can assume only positive values, larger or smaller than 1, corresponding to cooperative or anti-cooperative binding, respectively. It is worth recalling that both the Temkin and Hill models reduce to the non-cooperative standard Langmuir isotherm for *α_T_* = 0 and *n* = 1, respectively.

For the different Co loadings, the best-fit parameters according to both models are reported in panels (d–f) of Figure 4. At low Co concentration, both Fe and Co sites are consistent with a noncooperative ligation mechanism (*α_T_* ≈ 0, *n* ≈ 1). With increasing Co loading, a shift toward anti-cooperative behavior is observed for both sites, yielding *α_T_* = −0.09 and *n* = 0.64 for Fe and *α_T_* = −0.11 and *n* = 0.63 for Co, respectively. For the highest Co loading, Fe sites revert to a noncooperative response, whereas Co sites exhibit even stronger anti-cooperativity. The half-saturation pressure (*p*_0.5_) for the Fe sites is always lower than the corresponding value for the Co sites. Consistently, the zero-coverage adsorption energy values (*E*_*ads*_^0^) reveal a clear site preference for Fe in the early stages of the uptake. However, as the Co loading approaches the FeTPyP:Co = 1:1 ratio, a crossover occurs, with stronger CO ligation at Co rather than at Fe. This trend is fully consistent with the DFT calculations performed at CO saturation and full Co coverage, yielding a larger metal-molecule charge transfer for CO@Co (0.288 e^–^) with respect to Co@Fe (0.049 e^–^), respectively. In general, the *E*_*ads*_^0^ value indicates weak binding (for comparison, the adsorption energy of CO/Ir(111) is 1.4–1.9 eV),^72^ accounting for the observed reversibility of ligation at room temperature.

In the FeTPyP-Co system, we find clear evidence of anti-cooperativity (*n* < 1; *α_T_* < 0), despite the large distance between the nearest-neighbor ligation sites within the network (1.4 nm), which rules out the role of direct dipole–dipole-like lateral interactions. Indeed, for CO adsorption on bare metal surfaces, such as Ir(111),^60^ Pt(111),^73^ and Ru(0001),^59,74^ blueshifts of 30–80 cm^–1^ of the fundamental CO resonance are observed during the uptake at RT. These shifts are well explained by strong CO dipole–dipole coupling between adjacent molecules, separated by only a few Å. For the FeTPyP-Co system, STM imaging shows that the shortest distance between two adjacent metal sites available for ligation is larger by an order of magnitude: ∼1.9 nm between Fe–Fe and Co–Co (next-nearest-neighbor) sites and ∼1.4 nm between Fe–Co (nearest-neighbor) sites.^16^ At such distances, direct lateral interactions between ligands can be considered negligible, in line with previous observations for CO/FePc,^3^ CO/CoTPyP-Co,^23^ and CO/MnTPyP-Co.^24^ Indeed, the direct dipole–dipole interaction energy can be estimated from

6 $$U\left(r\right)=\frac{d^{2}}{4{\pi \varepsilon }_{0}r^{3}}$$

where *r* is the intermolecular distance, d the dipole moment, and *ε*_0_ the vacuum permittivity. Using *d* = *d*_CO_ = 0.112 D^3,23^ and *r* = 1.9 nm for Fe–Fe and Co–Co sites, and *r* = 1.4 nm for Fe–Co sites, the resulting interaction energies are *U* = 1.14 μeV and 2.8 μeV, respectively. These correspond to negligible wavenumber blue-shifts of the order of 0.01 cm^–1^. We also ruled out the possibility of Förster transfer of excitations.^59,60^ According to Förster’s model, an excited oscillator can exchange vibrational energy with an unexcited one by means of dipole–dipole coupling, which is, however, negligible in our case. The local-oscillator behavior of CO on Ru(001) has a breakdown at around 0.33 ML, due to the strong dipole–dipole coupling among adsorbates and the resulting vibrational energy delocalization,^59^ similarly to the case of CO/Ir(111).^60^ In those cases, the hot band is quenched, while the fundamental resonance broadens with increasing CO coverage, which are not compatible with our data. Therefore, the observed anti-cooperativity is most plausibly attributed to structural and electronic effects mediated by the heterostructure backbone, such as charge transfer and distortion of the organic platform induced by carbonylation, in close analogy to what has been demonstrated for similar systems like CoTPyP-Co and MnTPyP-Co.^23,24^

## 4 Conclusion

In this work, we presented a comprehensive picture of CO ligation in a graphene-supported FeTPyP–Co two-dimensional metal–organic network, investigated *in situ*, and the crucial interplay between structure, electronic configuration, and reactivity at single-atom sites was highlighted. Formation of the bimetallic network induces a Fe­(II)→Fe­(I) reduction, enabling CO adsorption at both Fe and Co sites at room temperature, which is not observed in the monometallic Fe system, thereby supporting an activation mechanism mediated by Co coordination. Combined IR-Vis SFG, AP-XPS, and DFT results reveal distinct adsorption geometries and charge-transfer characteristics at Fe and Co centers, together with ligation-induced changes in the metal electronic configuration and oxidation state. Quantitative adsorption isotherms reveal weak, reversible binding and uncover a mild anti-cooperative behavior, most likely governed by long-range electronic and structural effects mediated by the network backbone. These findings establish a biomimetic and tunable platform in which single-atom reactivity can be controlled via coordination engineering, offering new design principles for catalytic materials based on cooperative effects in low-dimensional systems.

## Supplementary Material



## Notes

Experimental and simulated STM images of the structure; SFG spectrum of the pristine FeTPyP layer without Co coordination; and XPS O 1*s* core-level spectra (PDF)
