# Supplementary material for: Ligation of Carbon Monoxide at Iron and Cobalt Single-Metal-Atom Sites: Competition for the +1 Oxidation State in a Surface-Confined Organic Network
Source: J Phys Chem C Nanomater Interfaces. 2026 Sep 15;130(38):13458–68. doi: 10.1021/acs.jpcc.6c04980 (PMC13618221; doi:10.1021/acs.jpcc.6c04980)
Supplement: Supplementary file 1 [file jp6c04980_si_001.pdf]

Supporting Information

Ligation of Carbon Monoxide at Iron and Cobalt Single Metal Atom Sites: Competition for the +1 Oxidation State in a Surface-Confined Organic Network.

A. Namar,[1] B. Roondhe,[2,3] S. Baronio,[1] M. De Col,[1] M. Jugovac,[1] M. Scardamaglia,[4] P. Giannozzi,[2,5] E. Vesselli[1,6,7,*]

[1]Department of Physics, University of Trieste, via A. Valerio 2 - 34127 - Trieste (Italy).

[2]Department of Mathematics, Computer Science, and Physics, University of Udine, Udine 33100, Italy

[3]Department of Materials Science and Engineering, KTH The Royal Institute of Technology, Stockholm, SE-100 44, Sweden

[4]MAX IV Laboratory, Lund University, Lund 22100, Sweden

[5]CNR-IOM – Istituto Officina dei Materiali, SISSA, Trieste 34136, Italy

[6]CNR-IOM - Istituto Officina dei Materiali, S.S. 14 km 163.5, Area Science Park – 34149 – Basovizza, Trieste (Italy).

[7]Center for Energy, Environment and Transport Giacomo Ciamician, University of Trieste – 34127 – Trieste (Italy)

[*]E-mail: evesselli@units.it

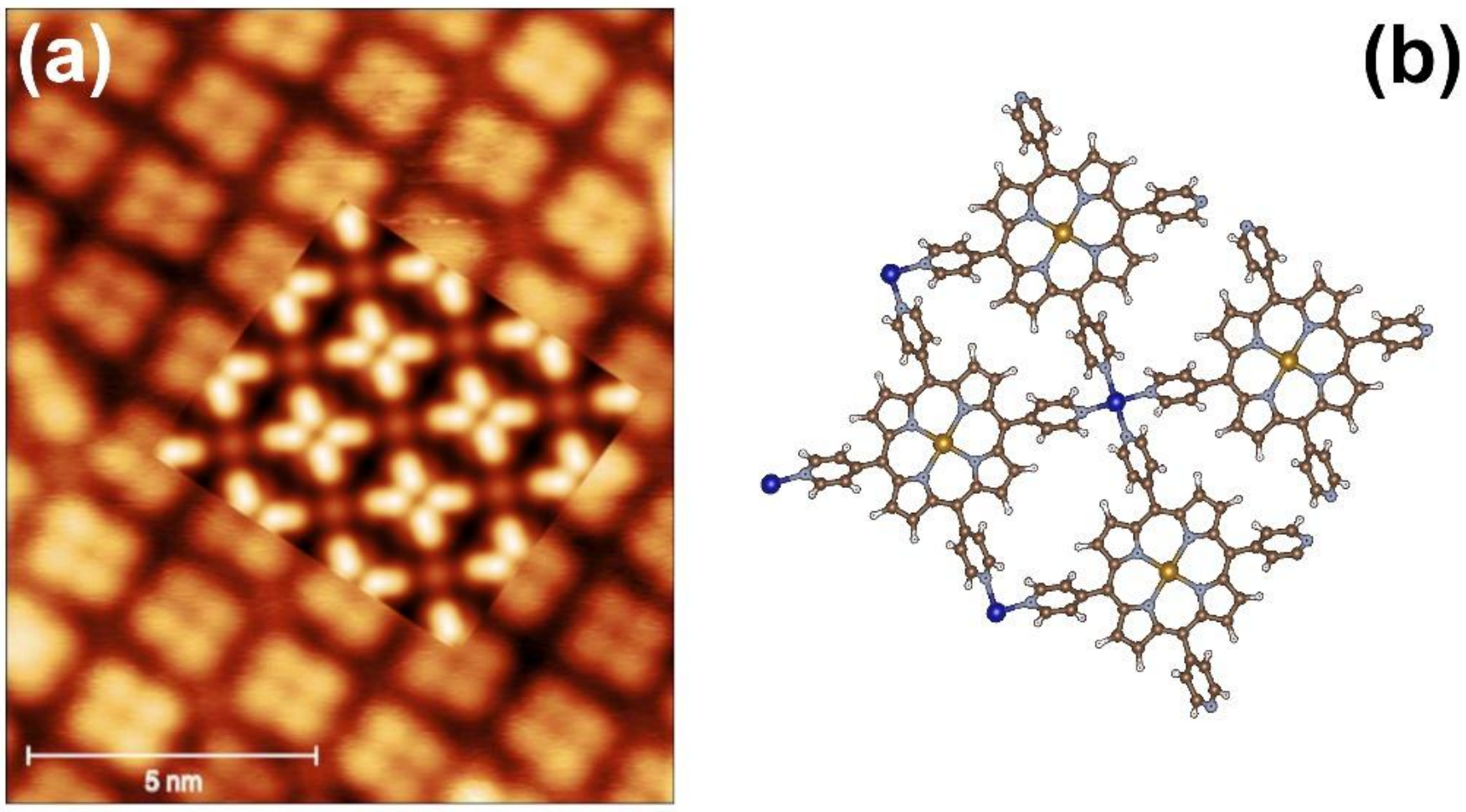

Figure S1. (a) Experimental STM image of the FeTPyP-Co/Gr/Ir(111) system at full Co coverage (Co:FeTPyP coverage ratio 1:1) yielding a (2 × 2) unit cell: the inset depicts the superimposed simulated image as obtained by the Tersoff-Hamann approach[1] from the structure optimized within the DFT calculation framework;[2] (b) DFT best structure geometry of the metalorganic network (underlying graphene is not shown for clarity) represented by a sticks-and-balls model (color map: Fe – gold, Co – blue, N – gray, C – brown, H – white). Further details about the geometric and electronic structures of the layer can be found in our recent work.[2]

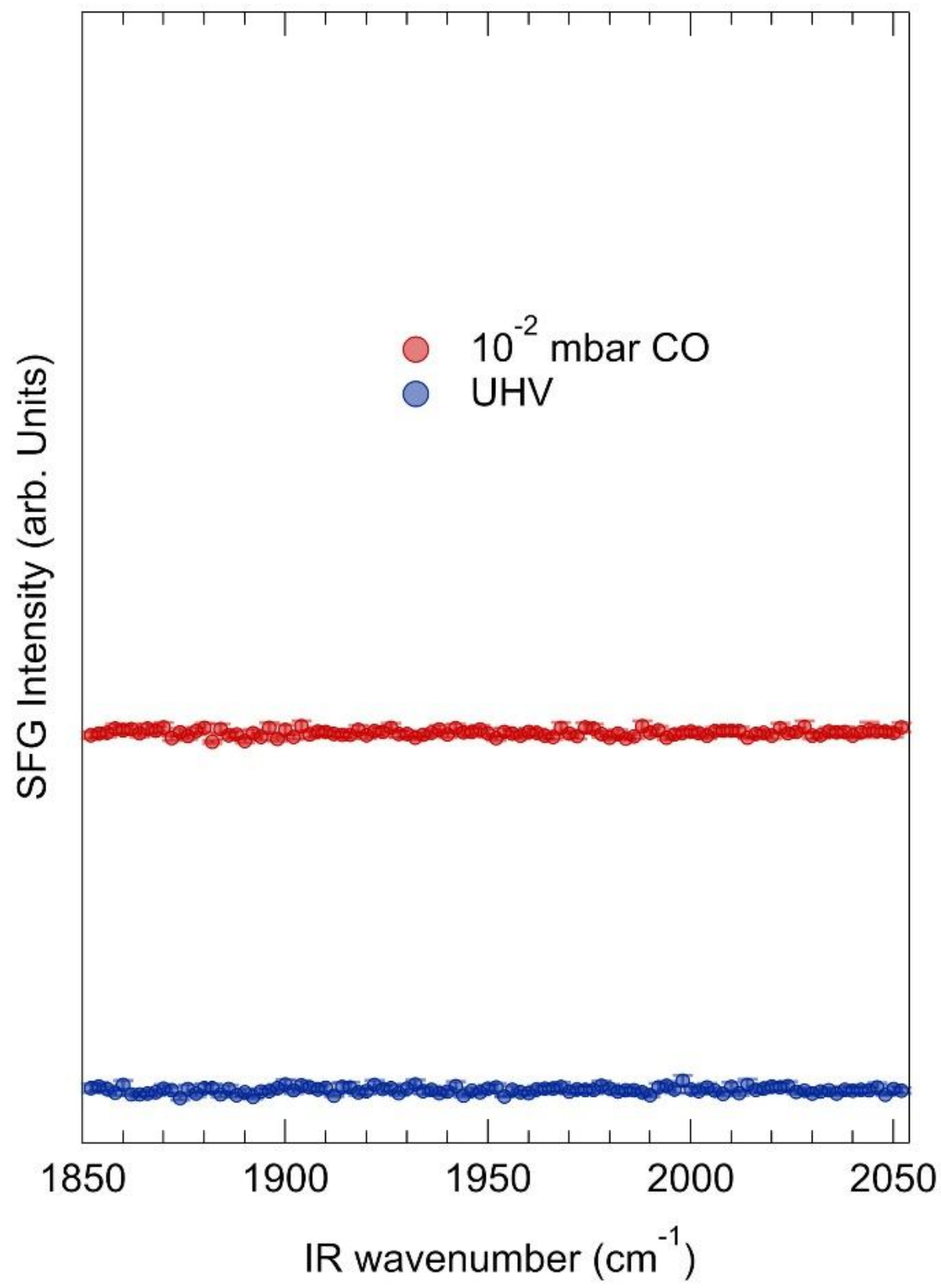

Figure S2. IR-Vis SFG spectra collected in the internal C-O stretch energy region at room temperature (*ppp* polarization combination) for the as-prepared FeTPyP/Gr/Ir(111) monolayer in UHV (bottom) and in 10^{-2} mbar CO. The layer is inert, at least up to this pressure value, showing no evidence for CO ligation at the Fe sites.

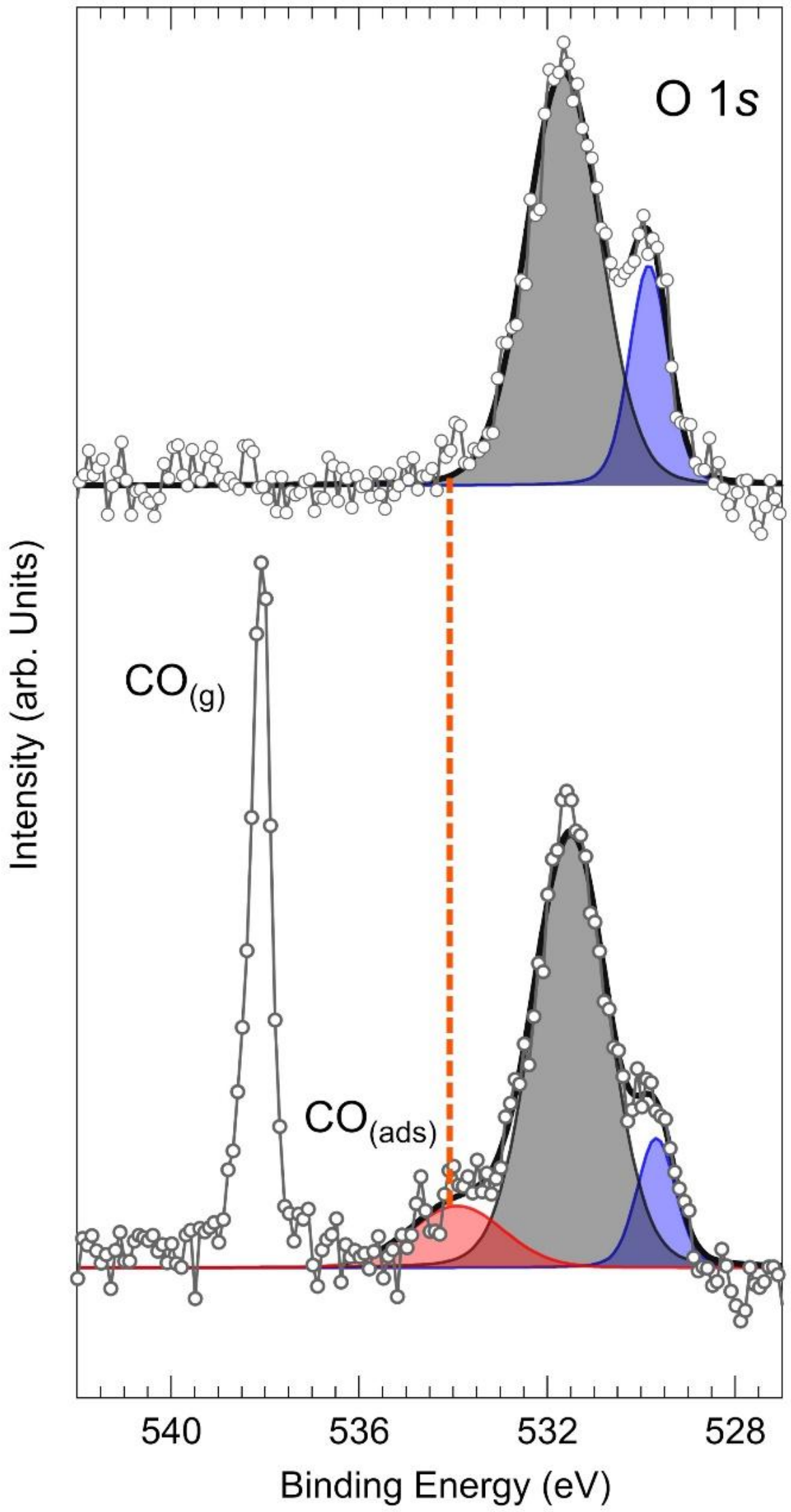

Figure S3. O 1*s* XPS spectra collected *in situ* in 10^{-2} mbar CO (bottom) and after recovering UHV conditions (top).

References.

(1) Tersoff, J.; Hamann, D. R. Theory of the Scanning Tunneling Microscope. *Phys. Rev. B* **1985**, *31* (2), 805–813. https://doi.org/10.1103/PhysRevB.31.805.

(2) Namar, A.; Roondhe, B.; De Col, M.; Baronio, S.; Jugovac, M.; Scardamaglia, M.; Frampton, E.; Vinogradov, N. A.; Giannozzi, P.; Vesselli, E. Stabilization of Strongly Reduced Fe(I)-like Single Atom Species Coordinated in a 2D Self-Assembled Metalorganic Network.
